# Supplementary material for: Discovery of a long-ranged charge order with 1/4 Ge1-dimerization in an antiferromagnetic Kagome metal
Source: Nat Commun. 2024 Jul 24;15:6262. doi: 10.1038/s41467-024-50661-x (PMC11269715; doi:10.1038/s41467-024-50661-x)
Supplement: Supplementary file 1 — Supplementary Information [file 41467_2024_50661_MOESM1_ESM.pdf]

Supplementary Information

Discovery of a long-ranged charge order with 1/4 Ge1-dimerization in an antiferromagnetic Kagome metal

Ziyuan Chen[1,†], Xueliang Wu[2,†], Shiming Zhou[1,†], Jiakang Zhang[1], Ruotong Yin[1], Yuanji Li[1], Mingzhe Li[1], Jiashuo Gong[1], Mingquan He[2], Yisheng Chai[2], Xiaoyuan Zhou[2], Yilin Wang[1,3,4], Aifeng Wang[2,*], Ya-Jun Yan[1,4,*], Dong-Lai Feng[1,3,4,5,6,*]

[1] Hefei National Research Center for Physical Sciences at the Microscale and Department of Physics, University of Science and Technology of China, Hefei, 230026, China

[2] Low temperature Physics Laboratory, College of Physics and Center of Quantum Materials and Devices, Chongqing University, Chongqing 401331, China

[3] National Synchrotron Radiation Laboratory School of Nuclear Science and Technology, and New Cornerstone Science Laboratory, University of Science and Technology of China, Hefei, 230026, China

[4] Hefei National Laboratory, University of Science and Technology of China, Hefei 230088, China

[5] Collaborative Innovation Center of Advanced Microstructures, Nanjing, 210093, China

[6] Shanghai Research Center for Quantum Sciences, Shanghai, 201315, China

Supplementary Note 1. Short-ranged CO in sample #1

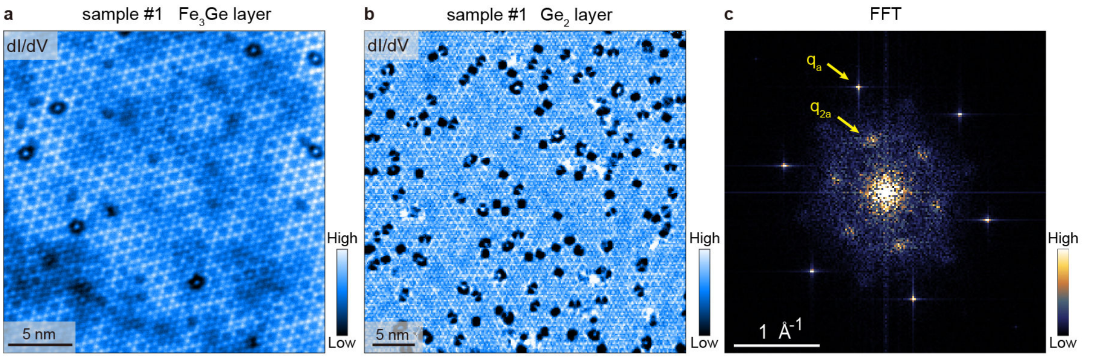

Supplementary Figure 1 | Short-ranged CO in sample #1. a,b, Typical *dI/dV* maps of the Fe_3Ge and Ge_2 layers of sample #1, showing a short-ranged CO in both surfaces. Panel **a** is adapted from ref. 1. **c,** FFT image of panel **a**, with the Bragg spots of underlying lattice and the CO spots labeled as q_a and q_{2a}, respectively. The q_{2a} spots are much more broadened than the q_a spots, further demonstrating the short-ranged behavior of the CO in sample #1. Measurement conditions: **a,** V_b = 0.3 V, I_t = 300 pA, ΔV = 30 mV; **b,** V_b = 0.4 V, I_t = 200 pA, ΔV = 5 mV.

Supplementary Note 2. More datasets for the long-ranged CO in sample #2

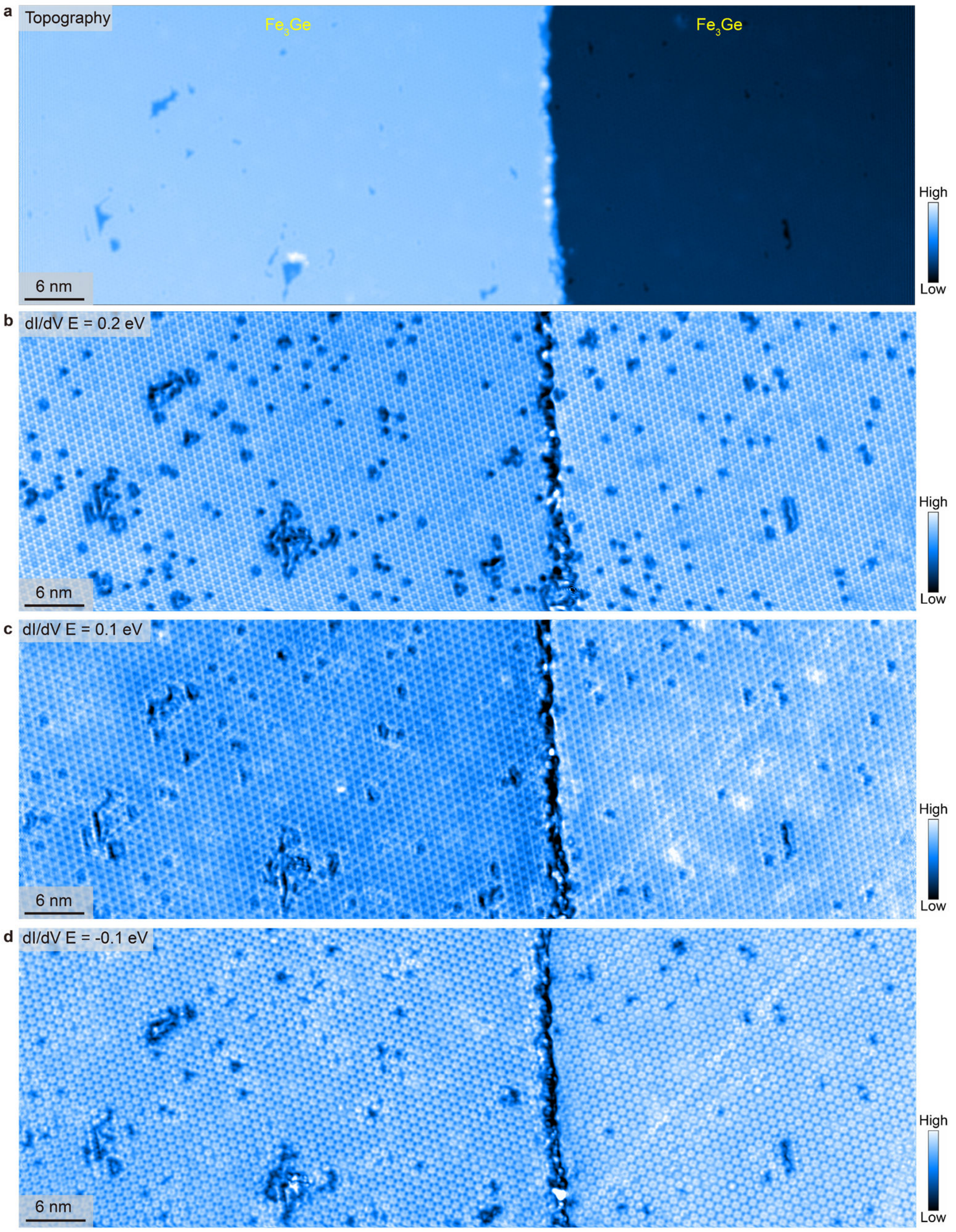

Supplementary Figure 2 | Topographic image and more *dI/dV* maps under various energies for a Fe_3Ge region. A long-ranged CO is obvious for all the maps, although the detailed CO patterns vary with energy. Measurement conditions: **a,** V_b = 0.3 V, I_t = 300 pA; **b,** V_b = 0.2 V, I_t = 300 pA, ΔV = 20 mV; **c,** V_b = 0.1 V, I_t = 300 pA, ΔV = 20 mV; **d,** V_b = -0.1 V, I_t = 300 pA, ΔV = 20 mV.

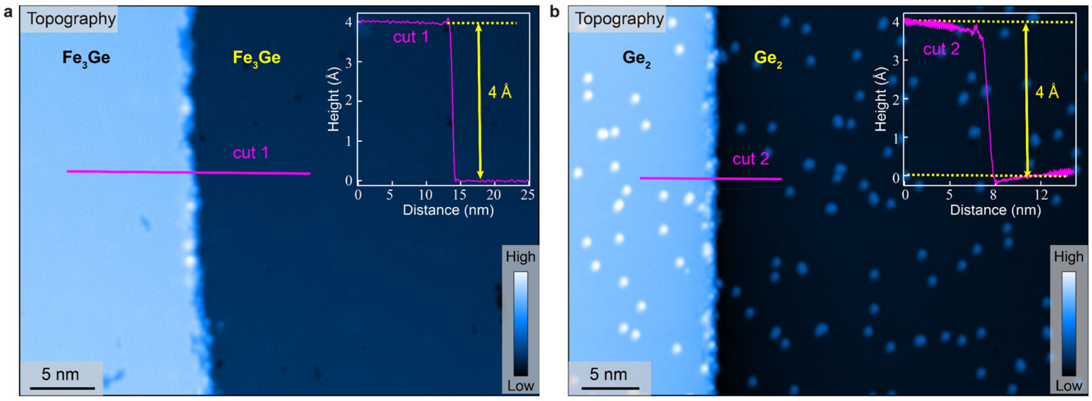

Supplementary Figure 3 | Topographic images of Fig. 2a,b. Insets: height profiles obtained along the magenta lines shown in panels **a**,**b**. The heights of the Fe_3Ge-Fe_3Ge and Ge_2-Ge_2 single-unit-cell-height steps are ~ 4 Å. Measurement conditions: **a,** V_b = 0.3 V, I_t = 300 pA; **b,** V_b = 0.2 V, I_t = 300 pA.

Supplementary Note 3. STM study on defect distribution in samples #1 and #2

In this part, we discuss the types and distribution of defects in samples #1 and #2 from the perspective of STM. Because STM lacks element resolution, one usually tries to judge the possible attribution of a defect from its crystallographic location and the influence pattern on the surrounding local density of states (LDOS) distribution[2,3]. Supplementary Fig. 4a shows the crystal structure of the 1 × 1 × 1 phase of FeGe, the Ge1-site defect, Ge2-site defect and Fe-site defect are illustrated by the black crosses in Supplementary Fig. 4b-d, and the green bars indicate the possible patterns of the LDOS distribution influenced by the defects, from the perspective of crystallographic symmetry. It's obvious that the defect pattern should be C_6-symmetric for Ge1-site defect, C_3-symmetric for Ge_2-site defect, and C_2-symmetric for Fe-site defect, respectively. Moreover, two degenerate C_3-symmetric states should exist for Ge_2-site defect, and three degenerate C_2-symmetric states should exist for Fe-site defect, as illustrated in Supplementary Fig. 4b-d.

Then we analyze STM topographic images and LDOS maps of multiple sample regions, and find mainly 7 types of defects on the Fe_3Ge layer and 2 types of defects on the Ge_2 layer. Supplementary Fig. 4e-h show the representative topographic images of a selected Fe_3Ge sample region measured under different bias voltages, it's obvious that different types of defects behave differently with varying bias voltages and are not always visible, as marked out by the dashed circles with different colors. It should be noted that almost all the defects are visible in the STM image measured at V_b = 1V, thus we use it to count the total density of defects.

Supplementary Fig. 4i-q display the detailed topographic images of nine types of defects.

1) Although the specific appearance is different, types 1-5 of defects on Fe_3Ge layer are all C_6-symmetric with their defect centers located at the Ge1-site. These defects are considered as Ge1 vacancies, substitutional defect at Ge1a-site, occupational disorder at Ge1b-site and so on, but the specific correspondence is difficult to determine simply by STM study.
2) Type 6 of defects on Fe_3Ge layer is C_3-symmetric, and has two orientations, which is more likely a Ge2-site defect when compared with Supplementary Fig. 4c. It should be a Ge2

vacancy or a substitutional defect at Ge2-site. One thing should be mentioned is that such C_3-symmetric defect is rarely observed on the Fe_3Ge layer in our STM study.

3) Type 7 of defect on Fe_3Ge layer is C_2-symmetric and has three orientations, which is more likely an Fe-site defect, such as an Fe vacancy or a substitutional defect. Such type of defect is also rarely observed in our STM study and was only found in a small Fe_3Ge region of sample #2.
4) Type 8 of defect is observed on the Ge_2 layer, it is C_6-symmetric with the defect center located at the Ge1-site, which might also be the Ge1-site defect seen on the Ge_2 layer.
5) Type 9 of defect on the Ge_2 layer is C_3-symmetric and has two orientations, which should be the Ge2-site defect.

One thing should be mentioned is that the abovementioned defect types on Fe_3Ge layer and Ge_2 layer may overlap, such as types 1-5 and type 8, type 6 and type 9, since STM measurements may include information of both the topmost layer and the layer underneath.

To simplify, we just count the total densities of Ge1-site, Ge2-site and Fe-site defects in multiple sample regions of samples #1 and #2, the results are shown in Supplementary Fig. 5. The total density of Ge1-site defects is reduced by approximately a third to a half in sample #2, from ~ 1.7% of the atomic proportion of Ge1-site in sample #1 to ~ 0.8% in sample #2. Fe-site defects are rarely observed and will not be discussed in detail here. The density of Ge2-site defect is as low as less than ~ 0.01 % of the atomic proportion of Ge2-site and is almost unchanged in both samples, which should have little influence on CO.

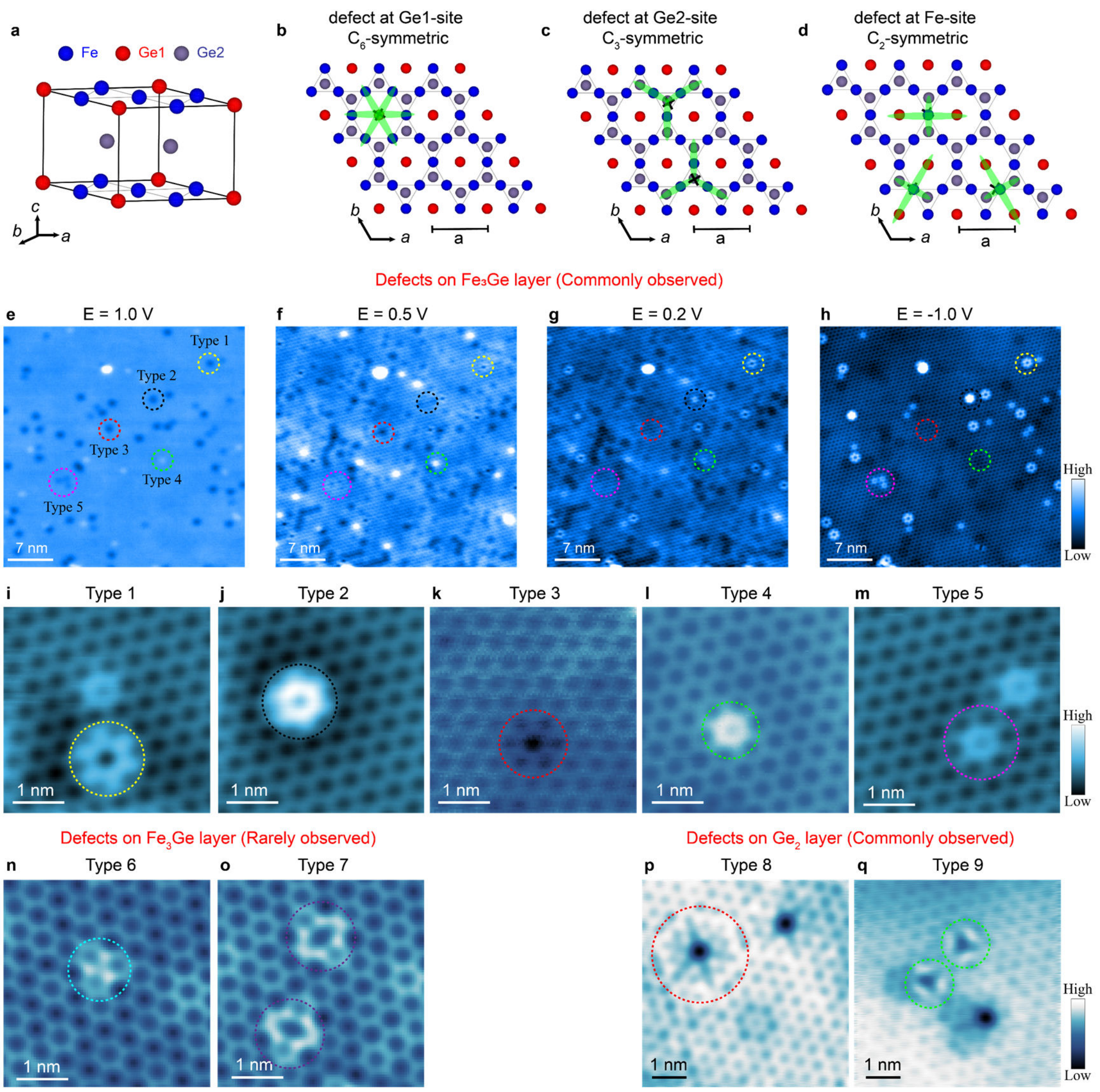

Supplementary Figure 4 | Different Types of defects observed in the Fe_3Ge and Ge_2 layers. **a,** Crystal structure of the 1 × 1 × 1 phase of FeGe. **b-d**, Sketches of crystallographic symmetries of defects located at different atomic sites. Defect locations are indicated by the black crosses, and their possible influence on LDOS is marked by the green bars. **e-h**, Typical topographic images for a selected Fe_3Ge region measured under different bias voltages. By comparing these images, we counted and classified all the defects into five types and marked them out by the dashed circles with different colors. **i-m**, Detailed topographic images of five types of Ge1-site defects, all are C_6-symmetric. **n**,**o**, Detailed topographic images of the other two types of defects that are occasionally observed in Fe_3Ge layer. The defects of types 6 and 7 are C_3- and C_2-symmetric, respectively, which might be the Ge2-site and Fe-site defects judging from their crystallographic symmetries. **p**,**q**, Detailed topographic images of the two types of defects observed on Ge_2 layer. The defects of types 8 and 9 are C_6- and C_3-symmetric, respectively, which might be the Ge1-site and Ge2-site defects judging from their crystallographic symmetries.

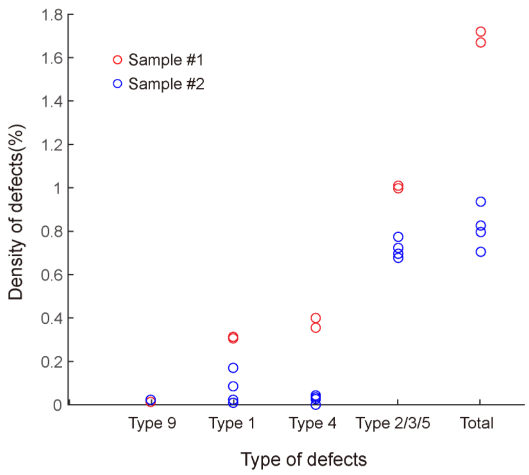

Supplementary Figure 5 | Statistical defect density in several sample regions of samples #1 and #2. Every red or blue hollow circle represents a sample region in samples #1 and #2, respectively. The density of Ge1-site defects (types 1-5) is reduced in sample #2, while the density of Ge2-site defect (type 9) is very low for both samples. The total density of all defects is reduced by approximately a third to a half in sample #2.

Supplementary Note 4. Detailed SCXRD results for samples #1 and #2

SCXRD measurements were carried out on both samples #1 and #2 at 300 K and 85 K, respectively. The diffraction patterns of these two samples are similar at 300 K, but vary obviously at 85 K, as displayed in Fig. 3, Supplementary Fig. 6 and Supplementary Fig. 7. At 85 K, new spots are observed for sample #2 and signal a structural modulation with a wave vector of (-0.5, 0.5, 0.5), while they are absent in sample #1.

By using Olex2 software with Shlex program[4,5], the corresponding crystal structures are solved and refined. Considering that the change of physical properties of FeGe by annealing is reversible[6,7] and the defect density observed in STM study is less than 2% as discussed above, we first adopt the full FeGe stoichiometry for simplicity, and the refined results are shown in Supplementary Table 1-6 and Supplementary Fig. 8. For both samples at 300 K, high quality refinements are achieved with the space group P6/*mmm*. The refined atomic coordinates are listed in Supplementary Table 2 and Table 5, and the corresponding crystal structure is sketched in Fig. 3c and Supplementary Fig. 8a-d, which is consistent with the previous report[8]. The same space group and atomic coordinates can also describe the diffraction patterns of sample #1 at 85 K (Supplementary Table 3). However, for the diffraction patterns of sample #2 at 85 K, a higher quality refinement is achieved with the space group P-6*m*2. The P-6*m*2 unit cell presented in Supplementary Table 4 is eight times larger than the primitive unit cell at 300 K, with the lattice doubling along all three lattice directions. The related atomic coordinates are listed in Supplementary Table 6, and the corresponding crystal structure is sketched in Fig. 3d and Supplementary Fig. 8e-h. The largest distortion occurs at the Ge1c sites in the Kagome layers, where the Ge1c atoms in the adjacent two layers dimerize and deviate from the Kagome layer by ~ 0.7 Å. Other atoms in the Kagome layer also undergo small distortions (< 5 pm), and are out-of-phase between adjacent Kagome layers. The other Ge1 atoms mainly distort along the *c*-axis, the Fe atoms are distorted both within the plane and along the *c*-axis. The distortion of Ge_2 layer is simpler, showing a deformed Kekulé-type distortion and is out-of-phase between adjacent Ge_2 layers.

Furthermore, we consider the effect of defects on the refinement. As shown in Supplementary

Figure 9a, when scrutinizing the SCXRD data collected at 300 K, we find that there is moderate residual electron density around Ge1b-site (Q peaks indicated by the black arrow), indicating obvious occupational disorders at Ge1-site. By adding such occupational disorders of Ge1-site, the refinements of the SCXRD data for samples #1 and #2 at 300 K are moderately improved, as listed in Supplementary Table 7. The related atomic coordinates are listed in Supplementary Table 8 and Table 9 for samples #1 and #2, respectively, and the corresponding crystal structure is sketched in Supplementary Fig. 9b. The obtained proportion of Ge1b-site to the total Ge1-site and their averaged distance from the Kagome plane are ~ 3% and ~ 0.973 Å for sample #1 and ~ 2% and ~ 0.865 Å for sample #2, respectively. Other types of defects, such as Ge2-site defects, don't show up in SCXRD data and the artificial introduction of a small amount of them has little effect on the refinements.

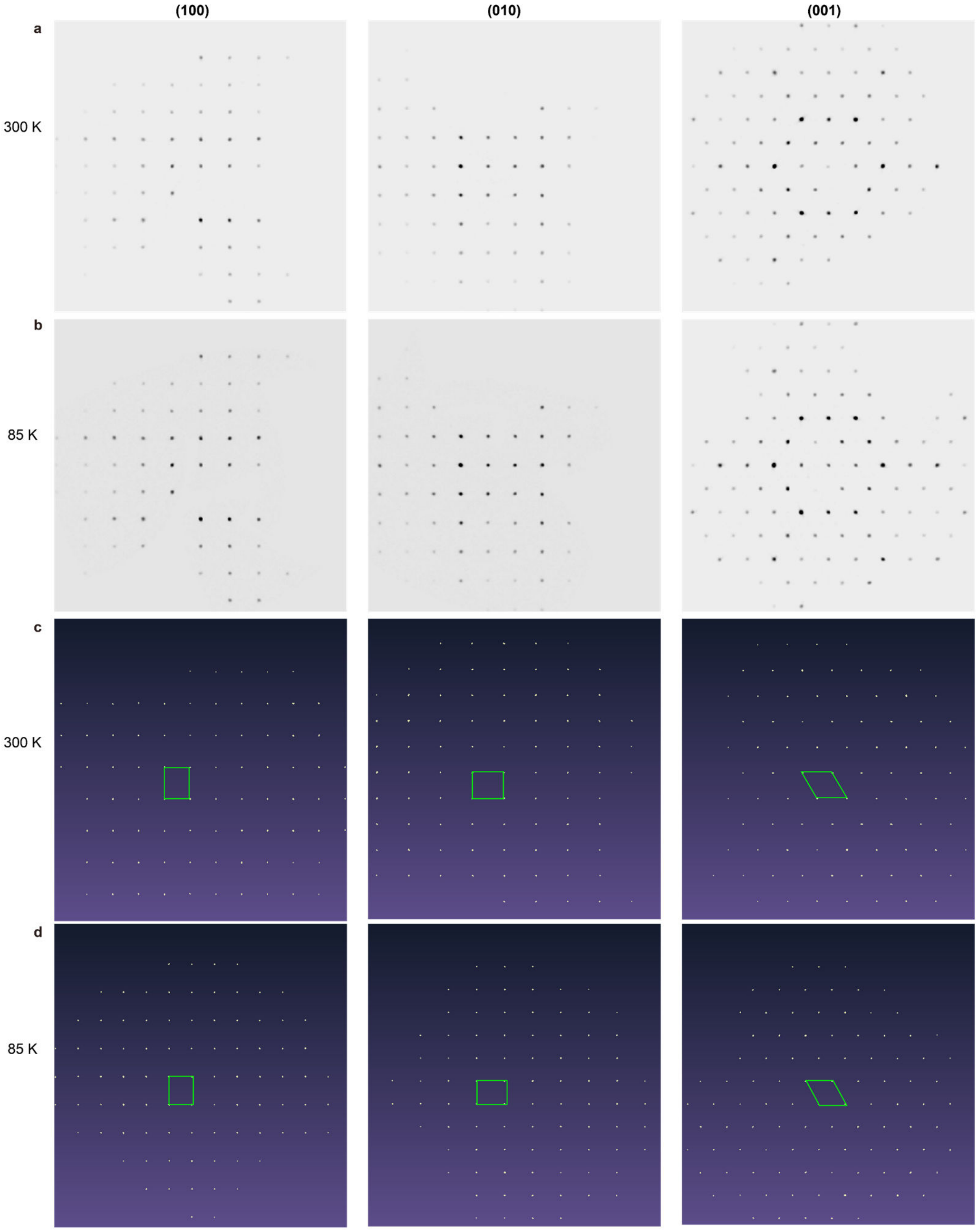

Supplementary Figure 6 | Diffraction patterns (a and b) and lattice peaks (c and d) along the *a*-, *b*- and *c*-axes for sample #1, measured at 300 K and 85 K, respectively. Unit cells are

indicated by the green rectangular and rhombic boxes. There are no obvious superstructure spots appeared at 85 K.

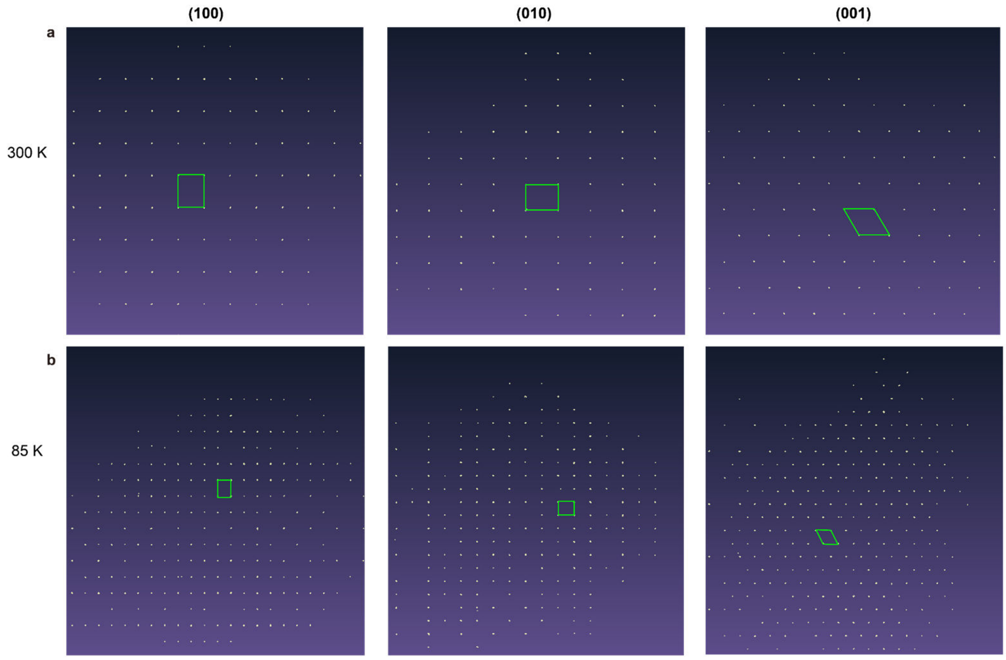

Supplementary Figure 7 | Lattice peaks along the *a*-, *b*-, and *c*-axes for sample #2 measured at 300 K and 85 K, respectively. Unit cells are indicated by the green rectangular and rhombic boxes. New spots appear at 85 K, signaling that the lattice doubles along all three lattice directions.

Supplementary Table 1. Crystal data and structure refinement for sample #1 at 300 K and 85 K.

**Temperature/K**	**300**	**85**
**Empirical formula**	FeGe	FeGe
**Formula weight**	128.44	128.44
**Crystal system**	hexagonal	hexagonal
**Space group**	P6/mmm	P6/mmm
**a/Å**	4.9956(8)	4.9859(4)
**b/Å**	4.9956(8)	4.9859(4)
**c/Å**	4.0606(7)	4.0471(4)
**α/°**	90	90
**β/°**	90	90
**γ/°**	120	120
**Volume/Å^3**	87.76(3)	87.131(17)
**Z**	3	3
**ρ_{calc}g/cm^3**	7.291	7.343
**μ/mm^{-1}**	37.158	37.426
**F(000)**	174	174
**Crystal size/mm^3**	0.16 × 0.14 × 0.14	0.16 × 0.14 × 0.14
**Radiation**	Mo Kα (λ = 0.71073)	Mo Kα (λ = 0.71073)
**2Θ range for data collection/°**	9.424 to 58.64	9.442 to 58.496
**Index ranges**	-6 ≤ h ≤ 5, -4 ≤ k ≤ 6, -5 ≤ l ≤ 4	-6 ≤ h ≤ 6, -6 ≤ k ≤ 6, -4 ≤ l ≤ 5
**Reflections collected**	505	591
**Independent reflections**	68 [R_{int} = 0.0335, R_{sigma} = 0.0218]	67 [R_{int} = 0.0408, R_{sigma} = 0.0200]
**Data/restraints/parameters**	68/0/8	67/0/9
**Goodness-of-fit on F^2**	1.280	1.214
**Final R indexes [I>=2σ (I)]**	R_1 = 0.0532, wR_2 = 0.1160	R_1 = 0.0569, wR_2 = 0.1371
**Final R indexes [all data]**	R_1 = 0.0532, wR_2 = 0.1160	R_1 = 0.0569, wR_2 = 0.1371
**Largest diff. peak/hole / e Å^{-3}**	4.91/-4.90	9.64/-4.79

Supplementary Table 2. Refined atomic coordinates and equivalent isotropic displacement (U_{eq}) parameters for sample #1 at 300 K.

Atom	*x*	*y*	*z*	U_{eq}	Occ.
**Ge1**	0	0	0	0.010	1.0
**Ge2**	1/3	2/3	1	0.005	1.0
**Fe1**	1/2	1/2	0	0.004	1.0

Supplementary Table 3. Refined atomic coordinates and equivalent isotropic displacement (U_{eq}) parameters for sample #1 at 85 K.

Atom	*x*	*y*	*z*	U_{eq}	Occ.
**Ge1**	0	0	0	0.011	1.0
**Ge2**	1/3	2/3	1	-0.003	1.0
**Fe1**	1/2	1/2	0	-0.001	1.0

Supplementary Table 4. Crystal data and structure refinement for sample #2 at 300 K and 85 K.

Temperature/K	300	85
Empirical formula	FeGe	FeGe
Formula weight	128.44	128.44
Crystal system	hexagonal	hexagonal
Space group	P6/mmm	P-6m2
a/Å	4.9948(6)	9.9552(8)
b/Å	4.9948(6)	9.9552(8)
c/Å	4.0534(5)	8.0817(7)
α/°	90	90
β/°	90	90
γ/°	120	120
Volume/Å^3	87.57(2)	693.64(13)
Z	3	24
ρ_{calc}g/cm^3	7.306	7.379
μ/mm^{-1}	37.237	37.610
F(000)	174.0	1392.0
Crystal size/mm^3	0.08 × 0.07 × 0.14	0.08 × 0.07 × 0.14
Radiation	Mo Kα (λ = 0.71073)	Mo Kα (λ = 0.71073)
2Θ range for data collection/°	9.424 to 58.664	6.91 to 58.65
Index ranges	$-6 \le h \le 6$, $-6 \le k \le 6$, $-5 \le l \le 5$	$-11 \le h \le 12$, $-12 \le k \le 13$, $-10 \le l \le 9$
Reflections collected	607	3493
Independent reflections	69 [R_{int} = 0.0329, R_{sigma} = 0.0158]	705 [R_{int} = 0.0256, R_{sigma} = 0.0206]
Data/restraints/parameters	69/0/9	705/0/55
Goodness-of-fit on F^2	1.275	1.092
Final R indexes [I>=2σ (I)]	R_1 = 0.0180, wR_2 = 0.0388	R_1 = 0.0317, wR_2 = 0.0724
Final R indexes [all data]	R_1 = 0.0180, wR_2 = 0.0388	R_1 = 0.0385, wR_2 = 0.0777
Largest diff. peak/hole / e Å^{-3}	2.29/-1.20	3.38/-1.68

Supplementary Table 5. Refined atomic coordinates and equivalent isotropic displacement (U_{eq}) parameters for sample #2 at 300 K.

Atom	*x*	*y*	*z*	U_{eq}	Occ.
Ge1	0	0	0	0.011	1.0
Ge2	1/3	2/3	1	0.007	1.0
Fe1	1/2	1/2	0	0.006	1.0

Supplementary Table 6. Refined atomic coordinates and equivalent isotropic displacement (U_{eq}) parameters for sample #2 at 85 K.

Atom	x	*y*	*z*	Ueq	Occ.
**Ge1c**	2/3	1/3	0.1656	0.006	1
**Ge1b**	0.1666	0.3332	0.2526	0.003	1
**Fe1a**	0.1661	0.0830	0.2530	0.001	1
**Fe1b**	0.4161	0.3343	0.2469	0.002	1
**Fe1c**	0.4171	0.5829	0.2530	0.002	1
**Ge2a**	0	0	1/2	-0.002	1
**Ge2b**	1/3	2/3	1/2	0.005	1
**Ge2c**	0.3440	0.1720	1/2	0.006	1
**Ge2d**	0.5043	0.4957	1/2	-0.002	1
**Ge2e**	0	0	0	-0.003	1
**Ge2f**	1/3	2/3	0	0.007	1
**Ge2g**	0.3268	0.1634	0	0.006	1
**Ge2h**	0.4967	0.5032	0	-0.003	1

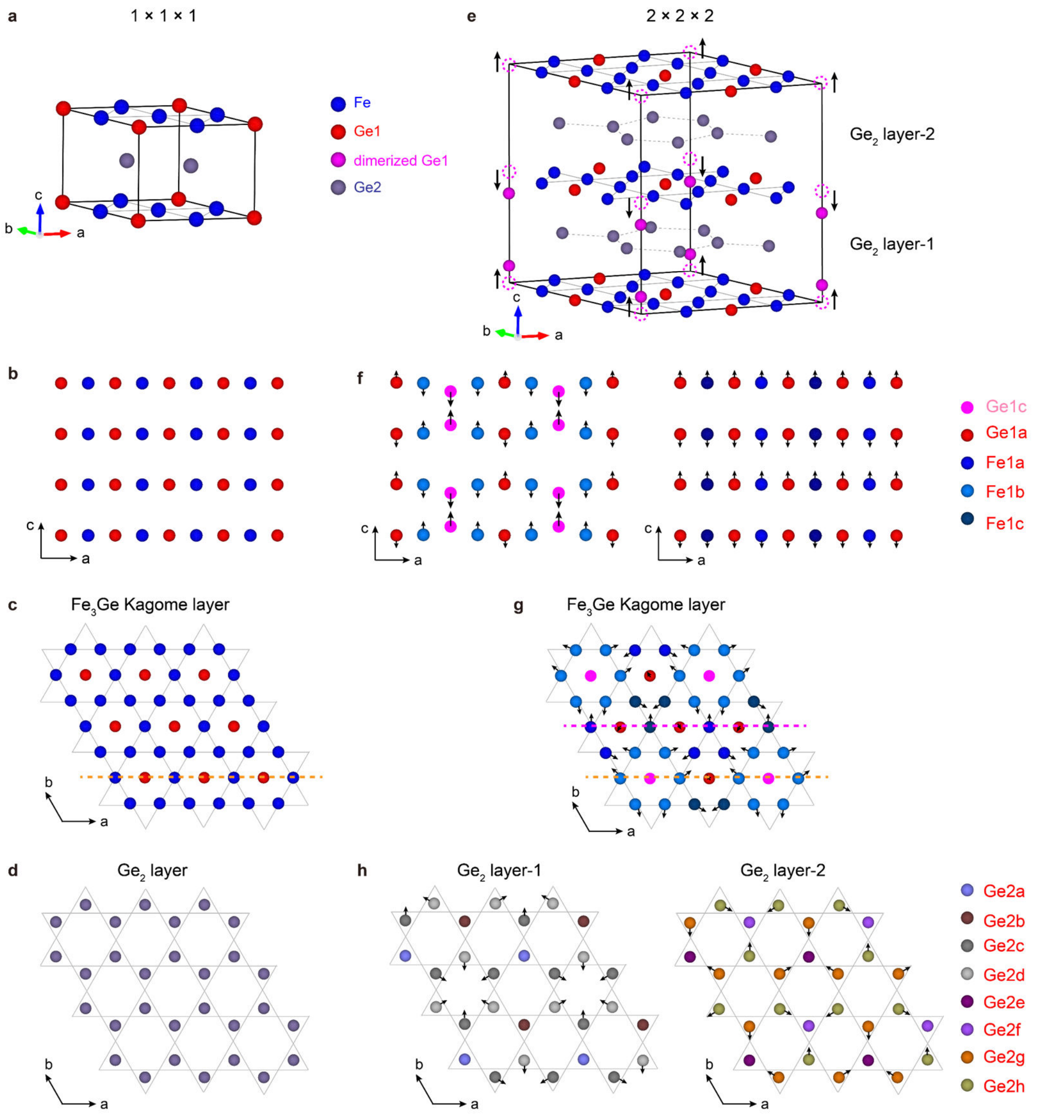

Supplementary Figure 8 | Detailed lattice distortion of the CO superstructure without considering the effect of defects. a-d, Lattice structure of FeGe at 300 K. Panel **b** shows the projection of (010) plane along the direction indicated by the orange dashed line in panel **c**. **e-h**, Detailed lattice structure of the CO superstructure measured at 85 K. Panel **f** shows the projections of (010) planes along the directions indicated by the orange and magenta dashed lines in panel **g**, exhibiting atomic distortions along the *c*-axis. **g,h,** In-plane atomic distortions in the Fe_3Ge and Ge_2 layers. The black arrows indicate the direction and magnitude of atom distortion.

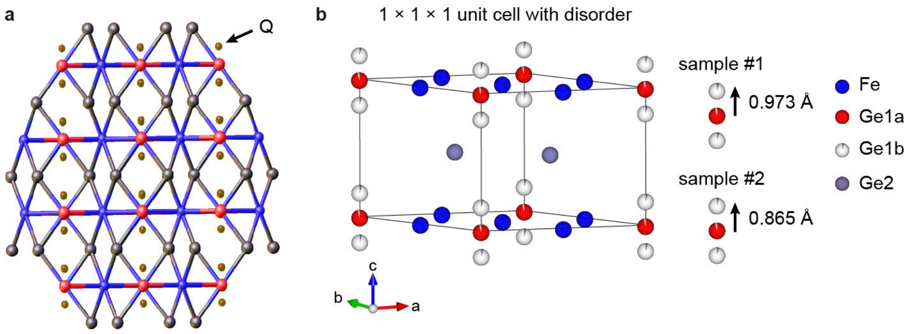

Supplementary Figure 9 | Refined lattice structure of FeGe at 300 K considering the occupational disorder of Ge1-site. a, Residual electron density (Q peaks) near Ge1-site at 300 K for sample #1. **b,** Corrected crystal structure of FeGe at 300 K, considering the occupational disorder of Ge1-site. The proportion of Ge1b-site to the total Ge1-site and the averaged distance from the Kagome plane are ~ 3% and 0.973 Å for sample #1 and ~ 2% and 0.865 Å for sample #2, respectively, as also sketched in the right panel of Fig. 5**e**.

Supplementary Table 7. Crystal data and structure refinement for samples #1 and #2 at 300 K with considering the occupational disorder of Ge1-site

Sample	#1	#2
Empirical formula	FeGe	FeGe
Formula weight	128.44	128.44
Crystal system	hexagonal	hexagonal
Space group	P6/mmm	P6/mmm
a/Å	4.9956(8)	4.9948(6)
b/Å	4.9956(8)	4.9948(6)
c/Å	4.0606(7)	4.0534(5)
α/°	90	90
β/°	90	90
γ/°	120	120
Volume/$Å^3$	87.76(3)	87.57(2)
Z	3	3
$\rho_{calc}g/cm^3$	7.291	7.306
μ/mm^{-1}	37.158	37.237
F(000)	174	174.0
Crystal size/mm^3	0.16 × 0.14 × 0.14	0.08 × 0.07 × 0.14
Radiation	Mo Kα (λ = 0.71073)	Mo Kα (λ = 0.71073)
2Θ range for data collection/°	9.424 to 58.64	9.424 to 58.664
Index ranges	$-6 \leq h \leq 5$, $-4 \leq k \leq 6$, $-5 \leq l \leq 4$	$-6 \leq h \leq 6$, $-6 \leq k \leq 6$, $-5 \leq l \leq 5$
Reflections collected	505	607
Independent reflections	68 [R_{int} = 0.0335, R_{sigma} = 0.0218]	69 [R_{int} = 0.0329, R_{sigma} = 0.0158]
Data/restraints/parameters	68/0/10	69/0/11
Goodness-of-fit on F^2	1.240	1.249
Final R indexes [I>=2σ (I)]	R_1 = 0.0463, wR_2 = 0.0978	R_1 = 0.0139, wR_2 = 0.0277
Final R indexes [all data]	R_1 = 0.0463, wR_2 = 0.0978	R_1 = 0.0139, wR_2 = 0.0277
Largest diff. peak/hole / e $Å^{-3}$	2.017/-4.064	0.634/-0.783

Supplementary Table 8. Refined atomic coordinates and equivalent isotropic displacement (U_{eq}) parameters for sample #1 at 300 K with considering the occupational disorder of Ge1-site

Atom	*x*	*y*	*z*	U_{eq}	Occ.
**Ge1a**	0	0	0	0.0091	0.971
**Ge1b**	0	0	0.240	0.0091	0.029
**Ge2**	1/3	2/3	1	0.0050	1.0
**Fe1**	1/2	1/2	0	0.0037	1.0

Supplementary Table 9. Refined atomic coordinates and equivalent isotropic displacement (U_{eq}) parameters for sample #2 at 300 K with considering the occupational disorder of Ge1-site

Atom	*x*	*y*	*z*	U_{eq}	Occ.
**Ge1a**	0	0	0	0.0101	0.977
**Ge1b**	0	0	0.214	0.0101	0.023
**Ge2**	1/3	2/3	1	0.0071	1.0
**Fe1**	1/2	1/2	0	0.0059	1.0

Supplementary Note 5. More datasets for CO disruption

Supplementary Fig. 10 shows the details of the 2 × 2 × 2 CO (Supplementary Fig. 10a-d) and the 1 × 1 × 1 phase (Supplementary Fig. 10e-h) obtained after CO disruption. By repeating the CO disruption process, the 2 × 2 × 2 CO disappears completely, leaving a pure 1 × 1 × 1 phase, as reflected more clearly in the FFT images (Supplementary Fig. 10f and 10h). Moreover, we have measured the topographic images and LDOS maps of several phase separation regions in both the Fe_3Ge and Ge_2 layers under a wide energy range, as shown in Supplementary Fig. 11. Under all these energies, the 2 × 2 × 2 CO is higher than the 1 × 1 × 1 phase in LDOS, but is lower in STM topography. This suggests that the STM topography truly reflects the relative height difference between these two phases, and the *c*-axis lattice parameter of the 1 × 1 × 1 phase is slightly larger than that of the 2 × 2 × 2 CO. Moreover, in all cases, the lattice of the 1 × 1 × 1 phase is slightly higher by ~ 6-9 pm than that of the 2 × 2 × 2 CO phase, as shown in Supplementary Fig. 11a3-e3,f3-i3.

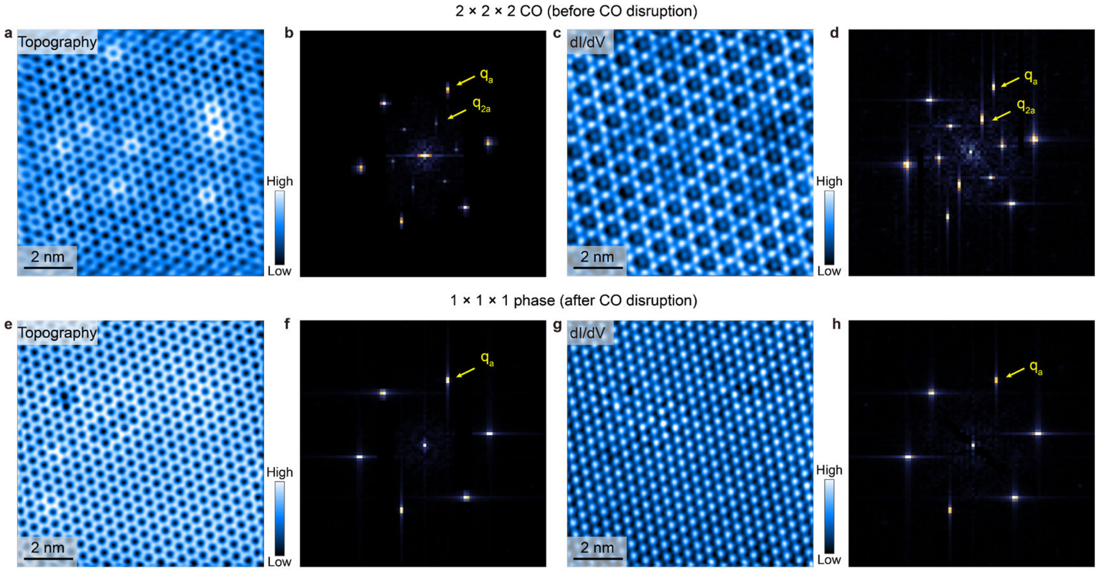

Supplementary Figure 10 | Details of the 2 × 2 × 2 CO and the 1 × 1 × 1 phase obtained after CO disruption. a-d, Typical topographic image, *dI/dV* map and corresponding FFT images of a selected Fe_3Ge region before CO disruption. **e-h,** Typical topographic image, *dI/dV* map and corresponding FFT images of the same Fe_3Ge region after CO disruption. The 2 × 2 × 2 CO disappears completely after CO disruption, leaving a pure 1 × 1 × 1 phase. Measurement conditions: **a,e,** $V_b = 0.2$ V, $I_t = 300$ pA; **c,g,** $V_b = 0.2$ V, $I_t = 300$ pA, $\Delta V = 30$ mV.

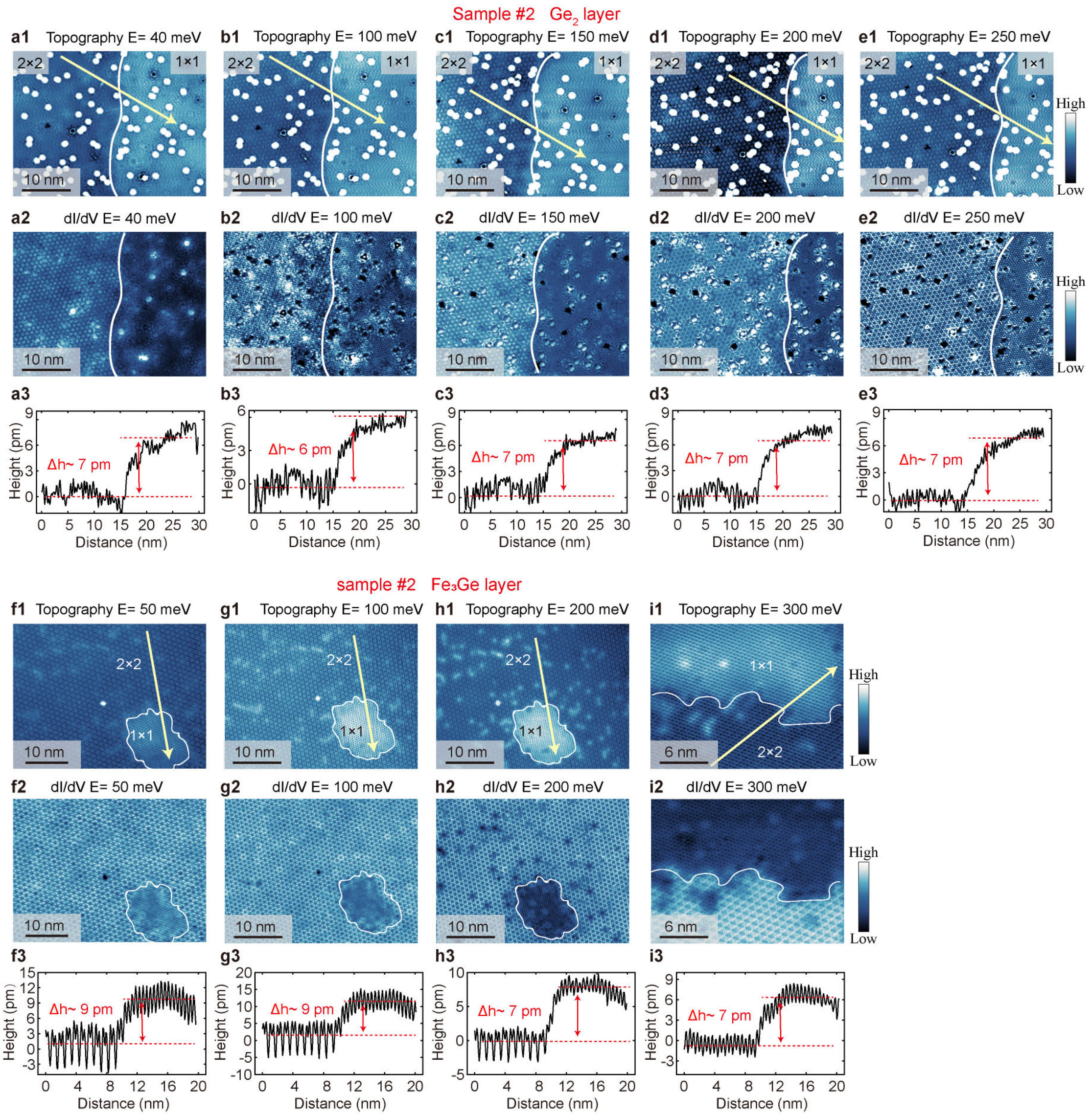

Supplementary Figure 11 | Comparison between the 2 × 2 × 2 CO and the 1 × 1 × 1 phase in both the Fe_3Ge and Ge_2 layers of sample #2. a1-e1, Typical topographic images measured under different energies, collected in a fixed sample region of Ge_2 layer consisting of both the 2 × 2 × 2 CO and the 1 × 1 × 1 phases. **a2-e2,** Corresponding *dI/dV* maps of panels **a1-e1**. **a3-e3,** Lattice line profiles taken along the yellow arrows in panels **a1-e1**. The height difference between the two phases, Δh, is marked out. **f1-i1,** Typical topographic images of the phase separated sample regions in Fe_3Ge layer measured under different energies. **f2-i2,** Corresponding *dI/dV* maps of panels **f1-i1**. **f3-i3,** Lattice line profiles taken along yellow arrows in panels **f1-i1**. The height difference between the two phases, Δh, is marked out.

Supplementary References:

1. Chen, Z. Y. et al. Charge density wave with strong quantum phase fluctuations in Kagome magnet FeGe. ArXiv: 2302.04490 (2023).
2. Yan, Y. J. et al. Surface electronic structure and evidence of plain s-wave superconductivity in $(Li_{0.8}Fe_{0.2})OHFeSe$. *Phys. Rev. B* **94**, 134502 (2016).
3. Novello, A. M. et al. Scanning tunneling microscopy of the charge density wave in 1T-$TiSe_2$ in the

presence of single atom defects. *Phys. Rev. B* **92**, 081101 (2015).

4. Dolomanov, O.V. et al. OLEX2: a complete structure solution, refinement and analysis program. *J. Appl. Cryst.* **42**, 339 (2009).
5. Sheldrick, G. M. A short history of SHELX. *Acta Cryst.* **A64**, 112 (2008).
6. Shi, C. F. et al. Disordered structure for long-range charge density wave order in annealed crystals of magnetic kagome FeGe. ArXiv: 2308.09034 (2023).
7. Wu, X. L. et al. Annealing tunable charge density wave order in a magnetic kagome material FeGe. ArXiv: 2308.01291 (2023).
8. Teng, X. K. et al. Discovery of charge density wave in a kagome lattice antiferromagnet. *Nature* **609**, 490 (2022).
